# Detection and discrimination of neutron capture events for NCEPT dose quantification

**DOI:** 10.1038/s41598-022-09676-x

**Published:** 2022-04-07

**Authors:** Andrew Chacon, Marissa Kielly, Harley Rutherford, Daniel R. Franklin, Anita Caracciolo, Luca Buonanno, Ilenia D’Adda, Anatoly Rosenfeld, Susanna Guatelli, Marco Carminati, Carlo Fiorini, Mitra Safavi-Naeini

**Affiliations:** 1 Australian Nuclear Science and Technology Organisation (ANSTO), Lucas Heights, Australia; 2 Centre for Medical Radiation Physics, University of Wollongong, Wollongong, Australia; 3 Faculty of Engineering and IT, University of Technology Sydney, Sydney, Australia; 4 Dipartimento di Elettronica, Informazione e Bioingegneria, Politecnico di Milano, Milan, Italy; 5 Istituto Nazionale di Fisica Nucleare (INFN), Sezione di Milano, Milan, Italy

**Keywords:** Cancer therapy, Radiotherapy, Particle physics, Nuclear physics

## Abstract

Neutron Capture Enhanced Particle Therapy (NCEPT) boosts the effectiveness of particle therapy by capturing thermal neutrons produced by beam-target nuclear interactions in and around the treatment site, using tumour-specific $^{10}$B or $^{157}$Gd-based neutron capture agents. Neutron captures release high-LET secondary particles together with gamma photons with energies of 478 keV or one of several energies up to 7.94 MeV, for $^{10}$B and $^{157}$Gd, respectively. A key requirement for NCEPT’s translation is the development of in vivo dosimetry techniques which can measure both the direct ion dose and the dose due to neutron capture. In this work, we report signatures which can be used to discriminate between photons resulting from neutron capture and those originating from other processes. A Geant4 Monte Carlo simulation study into timing and energy thresholds for discrimination of prompt gamma photons resulting from thermal neutron capture during NCEPT was conducted. Three simulated $300\times 300\times 300$ mm$^3$ cubic PMMA targets were irradiated by $^4$He or $^{12}$C ion beams with a spread out Bragg peak (SOBP) depth range of 60 mm; one target is homogeneous while the others include $10\times 10\times 10$ mm$^3$ neutron capture inserts (NCIs) of pure $^{10}$B or $^{157}$Gd located at the distal edge of the SOBP. The arrival times of photons and neutrons entering a simulated $50\times 50\times 50$ mm$^3$ ideal detector were recorded. A temporal mask of 50–60 ns was found to be optimal for maximising the discrimination of the photons resulting from the neutron capture by boron and gadolinium. A range of candidate detector and thermal neutron shielding materials were simulated, and detections meeting the proposed acceptance criteria (i.e. falling within the target energy window and arriving 60 ns post beam-off) were classified as true or false positives, depending on their origin. The ratio of true/false positives ($R_{TF}$) was calculated; for targets with $^{10}$B and $^{157}$Gd NCIs, the detector materials which resulted in the highest $R_{TF}$ were cadmium-shielded CdTe and boron-shielded LSO, respectively. The optimal irradiation period for both carbon and helium ions was 1 µs for the $^{10}$B NCI and 1 ms for the $^{157}$Gd NCI.

## Introduction

Neutron Capture Enhanced Particle Therapy (NCEPT) is a new type of radiotherapy first proposed by Safavi-Naeini et al.^1^. The principal aim of NCEPT is to boost the effectiveness of particle therapy (either proton or heavy ion therapy) by capturing the internally generated thermal neutrons produced in and around the treatment site as a byproduct of the particle therapy process, using a $^{10}$B or $^{157}$Gd-based neutron capture agent (NCA) which concentrates in cancer cells. Neutron capture by these (stable) isotopes results in the production of high-LET secondary particles (which are responsible for the main therapeutic benefits of both NCEPT and conventional neutron capture therapy) together with the emission of gamma photons at several specific energies^2–6^.

Planning, optimisation and delivery of NCEPT is more complex than for conventional particle therapy, since the internally-generated thermal neutron fluence depends on several factors, including treatment volume size and depth, and the cellular concentration of the NCA. A critical step in the translation of NCEPT is the development of in-vivo techniques for measurement of the total dose delivered to the patient, including both the ion dose and the dose due to neutron capture. Since virtually none of the primary radiation emerges from the patient, this must be performed indirectly via detection of prompt gamma radiation emitted at specific wavelengths during the boron or gadolinium neutron capture reactions. Therefore, it is necessary to develop an imaging system capable of localising these high energy prompt gamma photons and to develop techniques which allow the estimation of the dose resulting from boron and/or gadolinium neutron capture.

The prompt gamma spectrum produced during conventional particle therapy (i.e. in the absence of $^{10}$B or $^{157}$Gd in the target) has been previously reported in the literature for a variety of target materials and beam types, both experimentally and via simulation^7–9^; it includes a continuum, which decreases as an approximately exponential function of energy, and a number of spectral peaks due to deexcitation of oxygen or carbon nuclei to their ground state (notably at 4.44 MeV and 6.13 MeV, respectively)^7^. These excited states are very short-lived; the photons are mostly emitted within a few nanoseconds of the interaction between the target and the ion (hence the term *prompt gamma*). Several schemes to utilise these photons for range verification in particle therapy have been proposed^10–14^; the use of a temporal window to improve discrimination of prompt gamma photons from neutrons has also been explored by several authors^14–17^.

Neutron capture by $^{10}$B or $^{157}$Gd results in the emission of gamma photons whose spectra have well-defined peaks at 478 keV (in the case of $^{10}$B) and $\{$79.5 keV, 182 keV, 6.75 MeV, 7.86 MeV and 7.94 MeV$\}$ (in the case of $^{157}$Gd)^2–4,6,18^. In the context of particle therapy, the emission of the majority of these neutron-capture-associated gamma photons occurs some time after the arrival of the beam pulse, due to the time required for thermalisation after the neutrons are generated via fragmentation.

Acquisition and accurate identification of neutron-capture prompt gamma photons is challenging due to their high energy and the confounding presence of free neutrons and other particles in the radiation field. The ideal detector for this application should be able to reject neutrons (since the objective is to quantify neutron capture in the target rather than the detector) while also being able to clearly discriminate between prompt gamma photons emitted by the de-excitation of nuclear fragments created during irradiation and prompt gamma photons emitted during neutron capture.

In this work, we explore the timing and energy characteristics of prompt gamma photons originating from thermal neutron capture by $^{10}$B or $^{157}$Gd during irradiation with carbon or helium ions. For the first time we report unique signatures which can be used to discriminate between photons resulting from neutron capture and those originating from other processes. Finally, we estimate the achievable performance of both ideal and realistic detector models utilising these signatures for neutron capture discrimination, and identify suitable materials for shielding the detector from thermal neutrons without compromising gamma photon detection performance.

First, a Monte Carlo simulation model is constructed, in which a target phantom is irradiated by a spread out Bragg peak (SOBP) helium or carbon ion beam with a 60 mm depth range. The detectors are positioned proximal to the Bragg peak and orientated towards the region of interest, which minimises the number of neutrons interacting directly with the simulated detectors (since neutrons are predominately created with initial momentum parallel to the beam^14,17^). Three phantoms are used in the simulation: the first is a solid homgeneous block of poly(methyl methacrylate) (PMMA), while the second and third include pure $^{10}$B and $^{157}$Gd inserts located at the distal end of the SOBP. The energy and time of arrival (relative to the time of primary particle generation) of all neutrons entering the insert region of each phantom are scored; the time of arrival (also relative to the time of primary particle generation) and energy of photons which exit the phantom were also scored. Based on these results, a temporal mask and energy window suitable for neutron capture discrimination are designed.

The temporal mask and energy window are applied to all particles arriving at an ideal detector (perfect absorption, infinite energy resolution, no dead time) and the numbers of true and false positive and negative classifications (where the ground truth is the known origin of each detected event) are counted. True positive/negative detections are defined as those where the detection is correctly classified as neutron capture or non-capture; false positives are those detections which satisfy the timing and energy acceptance window but are not related to neutron capture events, and false negatives are those where the detection *was* related to neutron capture, but either energy has been lost due to scattering outside of the detector volume such that the photon falls outside of the acceptance windows, or the photon arrives early or late due to an unusually rapid or slow neutron thermalisation prior to capture.

For the final part of the study, realistic detector simulation models are used, and the same evaluation of true/false positive/negative detection is performed. Several alternative detector materials are evaluated, including direct-detection CdTe, CZT (for boron neutron capture) and LaBr$_3$:Ce, LSO:Ce, BGO and PbWO$_4$ scintillator-based detectors (for both boron and gadolinium neutron capture); these detectors are simulated both unshielded and with thin layers of several alternative thermal neutron shielding materials.

The remainder of this paper is divided into the following sections. The configuration used for the simulation is described in “Materials and methods” section. Results from the work are presented and discussed in “Results and discussion” section. A final summary and conclusions are presented in “Conclusion” section.

## Materials and methods

**Figure 1:**
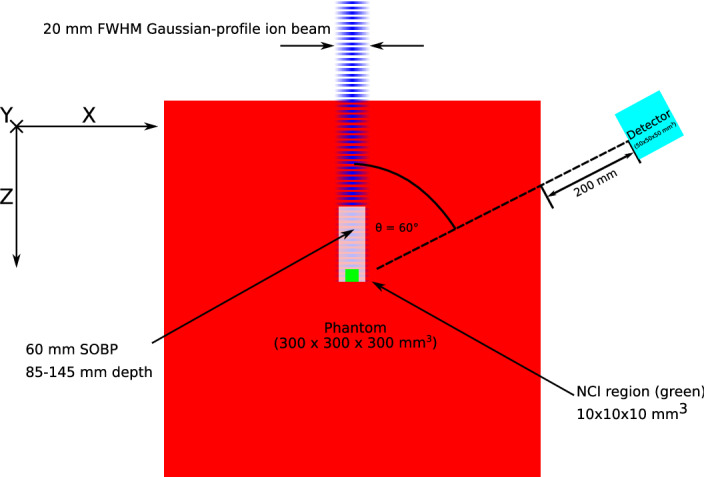
Schematic of the simulation configuration.

A Monte Carlo simulation model of an target phantom, ion beam and detector model was constructed in Geant4 as shown in Fig. 1^19^.

The simulated target phantoms are $300\times 300\times 300$ mm$^3$ PMMA cubes; the first is a homogeneous block of PMMA, while another two include a 10 mm cubic insert embedded within the PMMA phantom, with its centre at a depth of 140 mm (i.e. extending from 135 to 145 mm along the path of the beam, corresponding to the distal 10 mm of the spread-out Bragg peak) and centred laterally and vertically (in *x* and *y*), consisting of either pure $^{10}$B or $^{157}$Gd. The physical properties of the materials used in the simulations, including elemental compositions and density, are based on the library of standard materials defined by the National Institute of Standards and Technology (NIST)^20^.

Polyenergetic beams of $^{12}$C and $^{4}$He are synthesised with energy ranges of 225–294 MeV/u and 113–156 MeV/u, respectively, so as to create an approximately flat biological dose between depths of 85 mm and 145 mm in the phantom (the procedure used to create a flat biological dose is fully described in^1^). The ion beams are rotationally symmetric, with a 20 mm FWHM Gaussian beam profile, and are generated at the surface of the phantom, parallel to the *z*-axis of the phantom, with $10^8$ particles simulated for each beam and phantom combination. The energy deposited in the target phantom by each beam is scored as a function of depth across the full width at tenth maximum of the ion beam and normalised to the energy deposited at the entrance.

Geant4 version 10.2.p03 is used for all simulations, since it has been previously identified as providing the best agreement with experimental fragmentation measurements in particle therapy^19,21,22^. Electromagnetic interactions were modelled using the standard Geant4 physics option 3 model (G4EmStandardPhysics_option3), while the other physics models (including hadronic interactions) used in the simulation are listed in Table 1.

**Table 1:** Physics models used in all simulations.

Interaction	Energy range	Geant4 model
Radioactive decay	N/A	G4RadioactiveDecayPhysics
Particle decay	N/A	G4Decay
Hadron elastic	0–100 TeV	G4HadronElasticPhysicsHP
Ion inelastic	0–110 MeV	Binary Light Ion Cascade
	100 MeV–10 GeV	BIC
	9.99 GeV–1 TeV	FTFP
Neutron capture	0–20 MeV	NeutronHPCapture
	19.9 MeV–100 TeV	nRadCapture
Neutron inelastic	0–20 MeV	NeutronHPInelastic
Neutron elastic	0 eV–20 MeV	NeutronHPElastic
	20 MeV–100 TeV	hElasticCHIPS
Proton inelastic	0–9.9 GeV	Binary Cascade

It is known that the default Geant4 model for $^{157}$Gd neutron capture (as of 2022) has several deficiencies—in particular, while it reproduces the continuum component of the $^{157}$Gd neutron capture gamma spectrum accurately, many of the high-energy emission lines which have been observed experimentally are not correctly reproduced^18,23^. The specific issues with the standard Geant4 model and the impact of these on our $^{157}$Gd results are discussed in “Photons arriving at detector” section.

### Depth-dose profiles

Physical and biological depth-dose profiles for the SOBPs used in this work are generated by scoring energy deposited per unit mass in each voxel along the centre of the beam.

### Neutron and photon spectrograms

#### Neutrons entering NCI region

The energy and arrival time of neutrons entering the neutron capture insert region are recorded. Two-dimensional spectrograms characterising neutron arrival time (vertical axis) vs. kinetic energy (horizontal axis) are constructed. Time is measured relative to the instant at which the incident ion is generated at the surface of the phantom.

#### Photons arriving at detector

The energy and arrival time of photons entering the detector are recorded. Two-dimensional spectrograms characterising photon arrival time (vertical axis) vs. energy (horizontal axis) are constructed. Time is measured relative to the instant at which the incident ion is generated at the surface of the phantom. The duration of the prompt gamma emission period will be estimated by examining the spectrogram; this will be used to determine an appropriate mask interval which can be used to exclude these photons in “Detector material, irradiation period and shielding optimisation” section.

#### Neutrons arriving at detector

The energy and arrival time of neutrons entering the detector are recorded. Two-dimensional spectrograms characterising neutron arrival time (vertical axis) vs. energy (horizontal axis) are constructed. Time is measured relative to the instant at which the incident ion is generated at the surface of the phantom.

### Detector material, irradiation period and shielding optimisation

The second part of the study aims to determine the accuracy with which photons resulting from thermal neutron capture in the neutron capture insert can be distinguished from photons originating from other processes using several alternative detector models. Both idealised and physical detectors are simulated.

An ideal detector is modelled as a simple geometric volume which records the identity, creating process, time of arrival and energy of any particle entering the detector volume. The detector volume is cubic, with dimensions of 50 mm $\times \,$ 50 mm $\,\times \,$50 mm $^3$. The detector is positioned as shown in Fig. 1, with the normal vector of its front face oriented towards the centre of the distal edge of the SOBP region in the phantom. The front face of the detector is a distance of 47.32 cm from the centre of the distal edge of the SOBP, at an angle of $\theta = 60^\circ$ (about the vertical axis) relative to the ion beam.

The realistic detector models use the same geometry as the ideal detectors, and a range of different detector materials as listed in Table 2. LaBr$_3$, CZT and CdTe are suitable for detecting the gamma photons from $^{10}$B neutron capture, since they provide the highest energy resolution, while the higher densities of LSO:Ce, BGO and PbWO$_4$ are more appropriate for detecting the higher energy photons emitted during $^{157}$Gd neutron capture. It is assumed that several scintillator crystals or semiconductor detectors were stacked and logically/electronically coupled to form a single detector unit.

**Table 2:** Detector materials, including a range of scintillators and two direct-detection materials.

Material	Density (g/cm$^3$)	Yield (ph/MeV)	Decay time (ns)	Energy resolution (%)	Emission wavelength (nm)	Data source
LSO:Ce	7.10–7.40	30000	40	10.5	420	^24^
BGO	7.13	8000–10000	300	9.7	480	^25^
LaBr$_3$:Ce	5.06	63000	16–25	2.2–2.6	380–385	^26,27^
PbWO$_4$	8.28	400–500	6/30	36	440/530	^28,29^
CdTe	5.85	N/A (direct)	N/A (direct)	2.4	N/A (direct)	^30^
CZT	5.8	N/A (direct)	N/A (direct)	2–3	N/A (direct)	^27^

**Figure 2:**
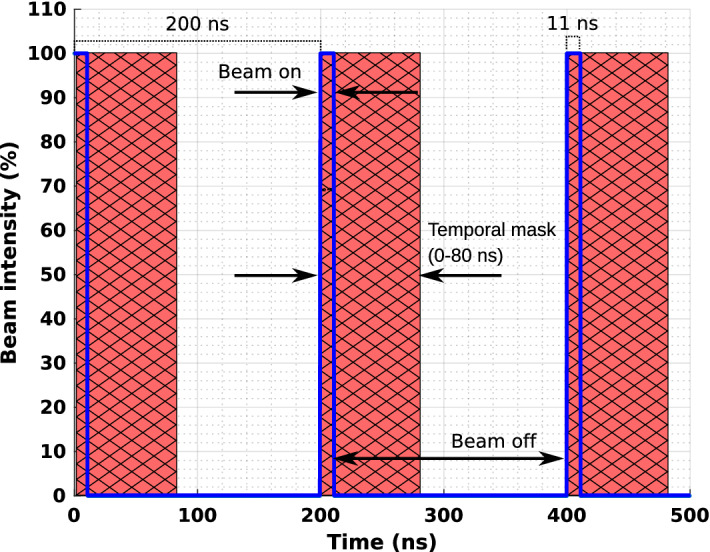
The micro-structure of the beam during the first 500 ns. This pattern is repeated for the entire irradiation period. The red hashed region indicates the range of the timing mask, during which events are rejected; events occurring outside of this range will be accepted.

Total irradiation periods of 1 µs, 10 µs, 1 ms, 10 ms and 100 ms are simulated. Over these intervals, the beam’s timing nanostructure is modelled as a pulse sequence with a period of 200 ns and a beam-on period of 11 ns (a duty cycle of 5.5%) as shown in Fig. 2^31,32^. A total of 10$^{9}$ primary particles are used for each simulation, with the particles injected periodically at a constant rate during each nano-spill; the particle injection rate dependent on the total irradiation period such that the total number of particles delivered during the macroscopic irradiation period is the same. For any particle which deposits energy in the detector, the particle type, creating process, time of arrival, total deposited energy, and location(s) of energy deposition in the detector are scored (see Fig. 1); all individual energy depositions resulting from multiple-interaction events (e.g. multiple Compton interactions) are summed. The optical scintillation process is not modelled in simulation. Modelling the optical scintillation process in Geant4 is possible but increases the execution time by several orders of magnitude, and therefore not implemented for this study. It is assumed that the area under the output pulse from the optical photodetector is proportional to the deposited energy.

Since the ion beams, target geometry and composition are the same as in the first part of this study, a phase space record of the prompt gamma photons generated in the previous simulation (including the energy, time, position and responsible process of particle creation within the phantom) is used to drive this simulation, substantially reducing the required simulation time.

Based on the results presented in the first part of this study, an energy window and temporal mask are defined to differentiate between neutron capture photons and photons unrelated to neutron capture in the NCI region. Energy deposited into the detector during the masked-out intervals was not scored. Eight different temporal masks are investigated:

- 0 ns—no mask applied;
- 11 ns—masking during beam-on period);
- 11 ns + $T_\mathrm {prompt}$—masking during beam-on period plus prompt gamma emission period ($T_\mathrm {prompt}$), to be determined based on the analysis described in “Photons arriving at detector” section; and
- 11 ns + $T_\mathrm {prompt} + T_\mathrm {neutron}$ = 30, 40, 50, 60, 70, 80 ns total— masking during beam-on period plus prompt gamma emission period plus a range of possible neutron capture emission periods.

The temporal masks are applied from the start of each nano-spill and extend over the total irradiation interval.

For each irradiation interval, ion beam (carbon and helium), and detector model (ideal plus each of the realistic models), detected events divided into four classes:

- True positives: neutron capture events which are detected during the temporal acceptance window and which fall within the defined energy acceptance window;
- True negatives: non-neutron capture events which are detected either outside of the temporal acceptance window or which do not fall within the energy acceptance window;
- False positives: non-neutron capture events which are detected during the temporal acceptance window and which fall within the defined energy acceptance window, and are therefore incorrectly classified as neutron capture events; and
- False negatives: neutron capture events which are detected either outside of the temporal acceptance window or which do not fall within the energy acceptance window, and therefore are incorrectly classified as non-neutron capture events. These may arise either due to unusually fast or slow neutron thermalisation (leading to long but low-probability tails in the temporal distribution of neutron captures), Compton scattering of the emitted photon in the phantom (resulting in a lower energy and an altered photon trajectory), Compton scattering in the detector (in the case of the realistic detector models) followed by the escape of the scattered photon (resulting in only part of the photon’s energy being deposited in the detector), or the detection of particles other than photons (e.g. fast neutrons, protons or other fragments) in the detector.

Following this classification, the following performance metrics are calculated:

- True positive sensitivity and false positive sensitivity, defined as the ratios of true positive and false positive detections to the total the number of photons reaching the detector; and
- Selectivity, defined using the the ratio of *true positives : false positives* ($R_{TF}$).

Using the true and false positive sensitivity performance metrics and the selectivity metric $R_{TF}$, a series of separate comparisons were performed to optimise different aspects of the detector design:

#### Comparison of detector materials

The performance metrics were evaluated for each of the different detector materials across a range of timing mask intervals with a fixed irradiation period. For the $^{10}$B NCI target, an irradiation period of 1 µs was chosen based on the results of the first part of the study, since after this period the relative numbers of 511 keV (created via annihilation of positrons resulting from the decay of short-lived $\beta ^+$-emitting fragmentation products) and 2.23 MeV photons (resulting from hydrogen neutron capture) begin to increase, which will make it difficult to discriminate between the background and the 478 keV photons from boron neutron capture. For the $^{157}$Gd NCI target, the irradiation period is less critical to absolute sensitivity and selectivity, therefore an arbitrary irradiation period of 1 ms was selected. Irradiation periods were subsequently optimised via the method described in “Comparison of irradiation periods” section.

#### Comparison of irradiation periods

The impact of (macroscopic) irradiation period on detector sensitivity and $R_{TF}$ for a range of timing mask intervals were evaluated for the best-performing materials identified in “Comparison of detector materials” section: firstly, for only the photons depositing energy in the detector, and secondly, for all particles depositing energy in the detector. The chosen detectors for this comparison (based on the results in “Comparison of detector materials” section) were the CdTe detector for the $^{10}$B NCI and the LSO detector for the $^{157}$Gd NCI.

#### Comparison of shielding materials

Utilising the best-performing detector materials identified in “Comparison of detector materials’Comparison of detector materials” section (CdTe for the $^{10}$B NCI and LSO for the $^{157}$Gd NCI), a range of different high thermal neutron cross-section front-face shielding materials were evaluated. The shield is a $50\times 50\times$1 mm layer of the evaluated material, applied to the front face of the detector only. For phantoms with a $^{10}$B NCI, (natural) gadolinium, cadmium and hafnium are evaluated as potential thermal neutron shielding materials, while for those with the $^{157}$Gd NCI, (natural) boron, cadmium and hafnium are evaluated.

## Results and discussion

### Depth-dose profiles

**Figure 3:**
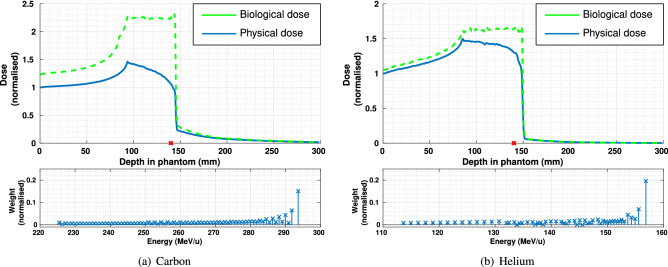
Depth-dose profiles (upper plot; blue = physical dose, green = biological dose) and energy spectra (lower plots) of polyenergetic carbon and helium ion beams; a red ‘X’ along the axis denotes the centroid of the NCI region.

The depth-dose profiles (normalised to entrance dose) and the energy weights used to generate the beams are shown in Fig. 3.

### Neutron and photon spectrograms

#### Neutrons entering NCI region

**Figure 4:**
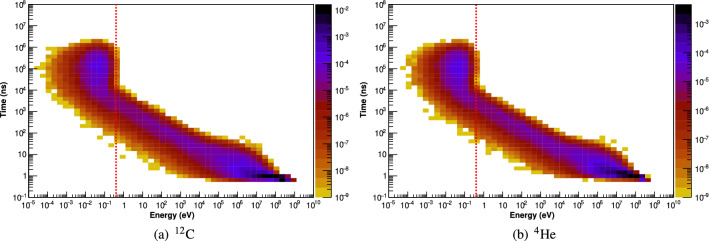
The arrival-time/energy spectrograms of neutrons entering the NCI region per incident particle following irradiation by polyenergetic carbon and helium ion beams. A vertical red dashed line is drawn at energy = 0.4 eV.

The two-dimensional spectrograms of neutron arrival time into the NCI region vs. energy are presented in Fig. 4 for carbon and helium ion beams. The energy and time of arrival of photons and neutrons entering the detector are scored and a photon arrival time (vertical axis) vs. energy (horizontal axis) spectrogram is produced. Arriving photons are distributed into 1000 linearly spaced energy bins (each 10 keV in width, from 0 to 10 MeV), and 100 logarithmically spaced arrival time bins (from 0.1 to 10$^{10}$ ns). Arriving neutrons are distributed into 130 logarithmically spaced energy bins (from 10$^{-10}$ to 10$^{3}$ MeV), and 100 logarithmically spaced arrival time bins (from 0.1 to 10$^{10}$ ns). Again, time is measured relative to the instant at which the primary particle is generated.

From the energy and timing distribution of the neutrons entering the NCI region (Fig. 4), it is apparent that arrival time of the neutrons depends on the energy of the neutron. Neutrons which are created with high initial kinetic energies must scatter multiple times to reach thermal equillibrium, which takes time. The neutron spectrograms for both carbon and helium ion beams exhibit similar characteristics, with thermal neutrons reaching the NCI region between $\sim$12 and 10$^6$ ns after creation. As a result of the delay between primary particle arrival and thermal neutron arrival time in the NCI region, it is expected that gamma emissions due to the thermal neutron capture process within the NCI will start to be observed between 12 and 10$^6$ ns after the onset of a beam pulse.

#### Photons arriving at detector

**Figure 5:**
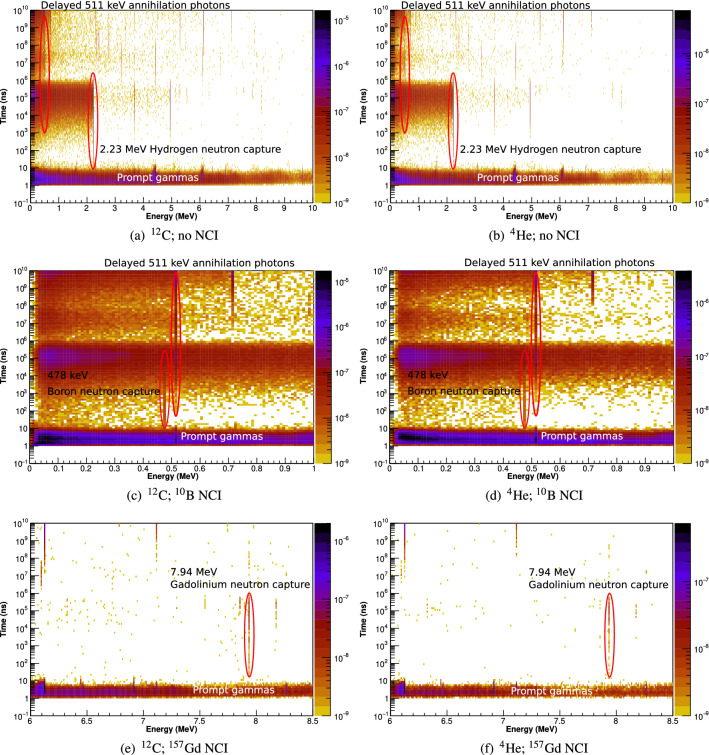
The energy/arrival-time spectrograms of photons entering the detector following an instantaneous irradiation by polyenergetic carbon and helium ion beams.

The energy and time of arrival of photons arriving at the detector volume are presented in Fig. 5.

In phantoms without the NCI (Fig. 5a,b for carbon and helium ion beams, respectively), all prompt (non-neutron-capture) photons arrive at the detector during the first 11 ns after the primary particle is generated. As previously discussed, thermalisation takes time, and as such, thermal neutrons principally appear during the 12 ns-10$^{6}$ ns temporal window (as can be seen from the 2.23 MeV hydrogen neutron capture line), with virtually none appearing outside of this window. Therefore, the use of the 12 ns-10$^6$ ns timing window for thermal neutron capture discrimination is justified. In phantoms both with and without the NCI, the 511 keV annihilation line starts to appear at approximately 10$^4$ ns and grows in intensity as time increases and the positron-emitting fragmentation products decay (binning is logarithmic in the temporal dimension).

In the phantoms with the $^{10}$B NCI (Fig. 5c,d for carbon and helium ion beams, respectively), the 478 keV line from $^{10}$B thermal neutron capture is visible during the period extending from approximately 12 ns to 10$^6$ ns, corresponding to the period when thermal neutrons are present in the phantom. However, the presence of the strong 511 keV positron annihilation signal nearby is a confounding factor in neutron capture discrimination, demanding high energy resolution from candidate detectors. The 2.23 MeV hydrogen neutron capture contributes to background at a broad range of lower energies, due to Compton scatter.

In the case of phantoms with the $^{157}$Gd NCI (Fig. 5e,f for carbon and helium ion beams, respectively), the 7.94 MeV emission line from $^{157}$Gd neutron capture is visible over the same time window as the $^{10}$B NCI line—from $\sim$12 to 10$^6$ ns. However, unlike the $^{10}$B case, there is no significant scatter background due to the absence of nearby decay or capture peaks.

The accuracy of our results for $^{157}$Gd are limited by the quality of the current Geant4 simulation model for $^{157}$Gd neutron capture. The Geant4 model is known to be unable to accurately reproduce all of the emission lines which have been observed experimentally^18,23^. However, we note that the emission line at 7.94 MeV which is seen in Fig. 5e,f (and which corresponds to the emission of the entire Q-value of the reaction as a single photon) has been observed experimentally, albeit with a low relative intensity compared to several other notable high-energy spectral peaks which are absent in our simulation. The remainder of our $^{157}$Gd analysis assumes that we are only able to detect the 7.94 MeV spectral line; however, in practice, we expect to be able to detect the additional spectral peak at 6.75 MeV as well. Since this line should be much more abundant compared to the 7.94 MeV peak, and also more readily detectable due to its lower energy, it is expected that the achievable true positive sensitivity will, in practice, be much greater than suggested by our results in “Detector materials, mask interval, irradiation duration and shield optimisation” section. A key planned future extension of this work is to integrate the Geant4 model published by the ANNRI-Gd Collaboration with our simulation model^4,18,33^.

#### Neutrons arriving at detector

**Figure 6:**
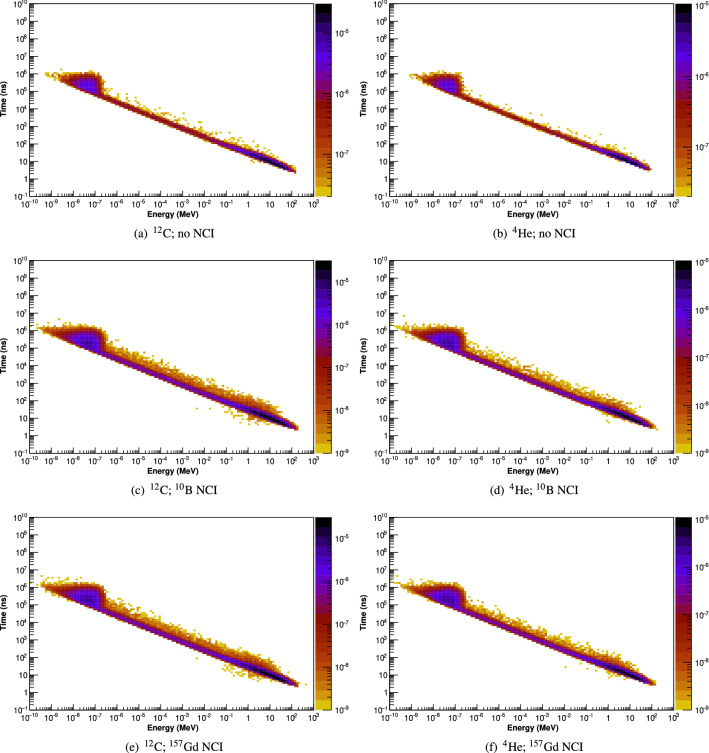
The energy/arrival-time spectrograms of neutrons entering the detector following irradiation by polyenergetic carbon and helium ion beams.

The energy and time of arrival of neutrons arriving at the detector are presented in Fig. 6.

In all phantoms, the time of arrival of neutrons at the detector depends on its energy (Fig. 6). For the purposes of this study, there are three energy and timing bands for neutrons arriving at the detector:

- Fast neutrons—energies above 1 MeV, with most neutrons arriving before 50 ns;
- Intermediate-energy neutrons—energies between 0.4 eV and 1 MeV, with most neutrons arriving between 50 and 10$^{4}$ ns; and
- Thermal neutrons—energies below 0.4 eV, which most neutrons arriving between 10$^{4}$ and 10$^{7}$ ns.

Fast and intermediate neutrons can potentially deposit enough energy in the detector to satisfy the energy window for neutron-capture prompt gamma detection, thereby creating false positives. Shielding against these neutrons is impractical, since stopping energetic neutrons at these requires large amounts of shielding material, which would also attenuate the gamma photons emitted during neutron capture processes in the target. The detection of fast neutrons can be greatly reduced by using a 50 ns timing mask post irradiation, since any fast neutrons arriving at the detector will do so within approximately 50 ns of the end of the beam pulse (providing that the detector itself does not become activated with long-lived radio-isotopes). Intermediate energy neutrons arrive at the detector in lower fluences and over a longer timescale compared to fast and thermal neutrons; while they potentially can deposit enough energy to trigger a false positive, they are much fewer in number compared to fast neutrons, and in any case, cannot be effectively blocked by shielding without compromising detector sensitivity.

### Detector materials, mask interval, irradiation duration and shield optimisation

An exhaustive search for the best timing mask and detector was undertaken; in summary, the CdTe detector with a 1 µs irradiation period was determined to be the optimal combination for the $^{10}$B neutron capture insert, since it provided the highest *true positives : false positives* ratio ($R_{TF}$), both for the unshielded detector and a detector shielded with 1 mm of cadmium. For the $^{157}$Gd neutron capture insert, the LSO:Ce detector with a 1 ms beam duration was the optimal combination, again providing the highest ($R_{TF}$) for both the unshielded detector and a detector shielded with 1 mm of boron.

For brevity, a subset of our results for carbon ion irradiation are presented here; specific plots comparing detector materials, irradiation duration and shielding materials are presented in “Detector materials–Shielding materials” sections. Corresponding results for helium ion irradiation are included in the Sections 1−3 of the Supplementary Material (Supplementary Figures 1, 2, 3); the best-performing detector materials with the helium ion beam were the same as for the carbon ion beam—CdTe (with optimal irradiation duration of 1 µs) and LSO:Ce (with optimal irradiation duration of 10 µs) for the $^{10}$B and $^{157}$Gd inserts, respectively.

Note that all plots in this section are shown with 95% confidence intervals ($\pm 2\sigma$), however in most cases the errorbars are too small to be visible under the markers.

#### Detector materials

**Figure 7:**
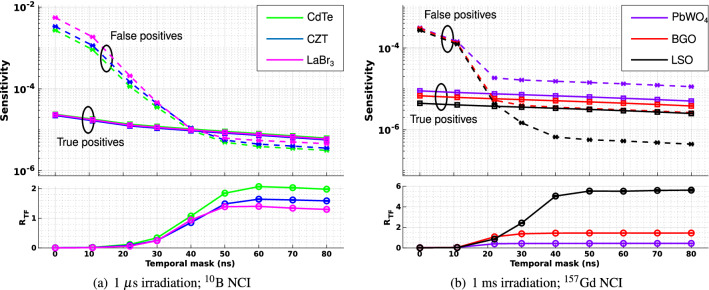
Sensitivity (upper plot) and R$_{TF}$ (lower plot) of events detected using different detector materials following irradiation by a carbon ion beam. For the $^{10}$B NCI, magenta denotes the LaBr$_{3}$ detector, green the CdTe detector and blue the CZT detector. For $^{157}$Gd NCI, red denotes the BGO detector, black the LSO detector and purple the PbWO$_4$ detector. In the upper plots in each subfigure, square markers ($\square$) joined by unbroken lines denote true positives, while cross markers ($\times$) joined by dashed lines denote false positives.

Figure 7 shows a comparison of the sensitivity and R$_{TF}$ of detectors with a fixed geometry and different detector materials irradiated by a carbon ion beam for fixed irradiation durations of 1 µs for the $^{10}$B NCI and 1 ms for the $^{157}$Gd NCI (a comparison of irradiation durations is discussed in the next subsection).

For the $^{10}$B NCI, both the highest true positive rate and lowest false positive rate were obtained with the CdTe detector, resulting in the highest $R_{TF}$ ($R_{TF} = 2.07\pm 0.01$) of all evaluated materials. The next-best performing material was CZT ($R_{TF} = 1.645\pm 0.009$) followed by LaBr$_{3}$ ($R_{TF} = 1.402 \pm 0.007$). For CdTe, the value of $R_{TF}$ is maximised when the mask interval reaches 60 ns, beyond which it declines slightly (Fig. 9). True-positive sensitivity is better at at 50 ns than at 60 ns, while $R_{TF}$ is only slightly lower (note the logarithmic scale of the upper plots); therefore 50 ns may be a better mask interval for the $^{10}$B insert, depending on whether the priority is sensitivity or selectivity.

For the $^{157}$Gd NCI, the discrimination problem involves a different set of trade-offs compared to the $^{10}$B NCI case. With the $^{157}$Gd NCI there is a near-total absence of scatter from photons with energy above the acceptance window, which implies that all false positives will be a result of neutron interactions within the detector. The PbWO$_4$ detector had the highest absolute sensitivity to true positives due to its high density, however, for all timing masks, the false positive rates were higher than the true positive rates, leading to a very poor maximum $R_{TF}$ compared to the other materials (0.442±0.002). If the detector could be sufficiently shielded against intermediate energy neutrons to reduce the rate of false positives, it might be a more competitive choice of detector material in cases where absolute sensitivity is prioritised. If, instead, selectivity is prioritised, then LSO is the best-performing detector material by a factor of almost 4 over the the next-best material ($R_{TF} = 5.52\pm 0.06$ compared with $R_{TF} = 1.454\pm 0.008$ for BGO); however, its absolute true positive sensitivity is the lowest amongst the evaluated materials. The low false positive rate of LSO is due to the lack of neutron capture emission lines in the energy acceptance window for any of of its constituent elements/isotopes. The much better selectivity of LSO compared to the other materials make it the preferred material for neutron capture discrimination for a $^{157}$Gd NCI. Finally, for the $^{157}$ NCI, $R_{TF}$ reaches its maximum value for all evaluated materials when the temporal mask is set to 50 ns, with no further improvements in $R_{TF}$ and a continuing decline in sensitivity obtained for mask intervals longer than 50 ns.

Corresponding results for helium ions are presented in Section 1 of the Supplementary Material (Supplementary Figure 1); the best materials were the same for helium ions as for carbon ions. All remaining simulations employ the best-performing materials for each NCI type.

#### Total irradiation period

**Figure 8:**
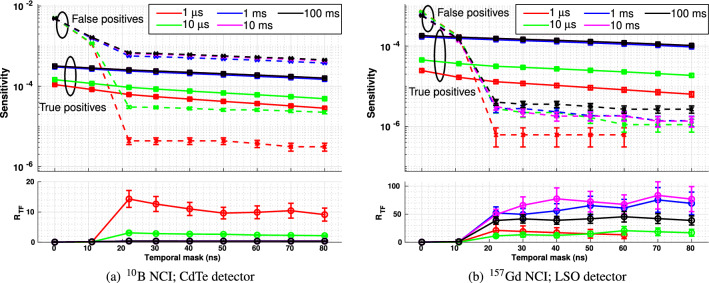
Sensitivity (upper plot) and R$_{TF}$ (lower plot) for **photon detections only** following target irradiation by a carbon ion beam as a function of temporal mask duration for a range of different irradiation periods. In all plots, the red markers denote a total irradiation time of 1 µs, green 10 µs, blue 1 ms, magenta 10 ms and black 100 ms. In the upper plots in each subfigure, square markers ($\square$) joined by unbroken lines denote true positives, while cross markers ($\times$) joined by dashed lines denote false positives.

**Figure 9:**
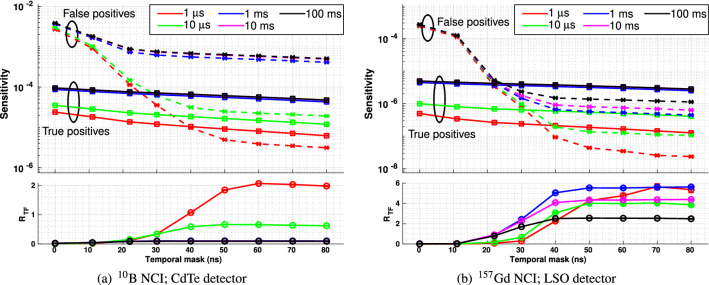
Sensitivity (upper plot) and R$_{TF}$ (lower plot) for **all detected events** following target irradiation by a carbon ion beam as a function of temporal mask duration for a range of different irradiation periods. In all plots, the red markers denote a total irradiation time of 1 µs, green 10 µs, blue 1 ms, magenta 10 ms and black 100 ms. In the upper plots in each subfigure, square markers ($\square$) joined by unbroken lines denote true positives, while cross markers ($\times$) joined by dashed lines denote false positives.

Figure 8 illustrates the performance (in terms of sensitivity and R$_{TF}$ vs. temporal mask duration) of physical CdTe and LSO detectors with $^{10}$B and $^{157}$Gd NCIs, respectively, with perfect neutron shielding. Fig. 9 shows the same measurements performed with detectors with no neutron shielding.

For the perfectly-neutron-shielded case, an irradiation period of 1 µs for the $^{10}$B NCI resulted in the highest detector selectivity ($R_{TF}$ peaking at 14 for a temporal mask interval of 22 ns and remaining above 9 for all longer mask intervals). However, absolute sensitivity was lowest for this irradiation interval for all temporal mask periods. Irradiation intervals of 1 ms-100 ms cannot be used at all since in each case $R_{TF}<< 1$. This is largely due to the presence of many photons with energies above the acceptance window arriving at the detector between 1 µs and 100 ms after irradiation. When these photons interact with detector via Compton scattering, they may deposit energy within the acceptance window, contributing greatly to the false positive rate. This problem can be largely eliminated with a shorter irradiation duration.

For the $^{157}$Gd NCI, both selectivity and sensitivity are much higher than for $^{10}$B, with irradiation intervals of 1 ms-100 ms resulting in approximately equal-highest true positive detection rates which greatly exceed the false positive detection rate - leading to $R_{TF} > 30$ in all of these cases (10 ms being optimal). As for the $^{10}$B NCI, $R_{TF}$ is maximised when the temporal mask exceeds 22 ns. There are very few photons with energies in excess of the acceptance window, and thus the impact of Compton scatter is on the false positive rate is negligible.

A comparison of the perfectly-neutron-shielded detector with the unshielded detector provides a clear illustration of the effect of neutrons on detector performance. Any neutrons which which deposit energy in the detector will do so within the first 60 ns (see Fig. 6), increasing the false negative count for this period and decreasing R$_{TF}$. In Fig. 9, the highest selectivity ($R_{TF}$) for carbon ion irradiation of a $^{10}$B NCI is still achieved with the 1 µs irradiation period, however its value reaches a maximum when the temporal mask period is greater than 60 ns, since the false negative rate continues to decline rapidly up to this point. For the $^{157}$Gd insert, unshielded detector selectivity is maximised with a 1 ms irradiation period, with $R_{TF}$ reaching a maximum value after 50 ns; this irradiation period also provides the highest absolute sensitivity to true positives.

In all cases, the selectivity of the unshielded detector is much lower than the corresponding perfectly-neutron-shielded case.

### Shielding materials

**Figure 10:**
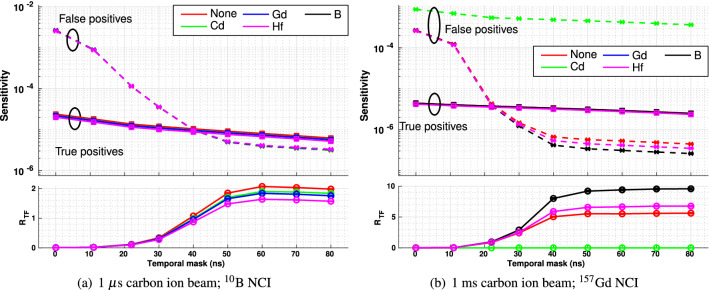
Sensitivity (upper plot) and R$_{TF}$ (lower plot) for events recorded in a detector shielded by different materials with high thermal neutron cross-section, following target irradiation by a carbon ion beam. In all plots, the red markers denote an unshielded detector, green cadmium shielding, blue gadolinium shielding ($^{10}$B NCI only), black boron shielding ($^{157}$Gd NCI only), and magenta hafnium shielding. In the upper plots in each subfigure, square markers ($\square$) joined by unbroken lines denote true positives, while cross markers ($\times$) joined by dashed lines denote false positives.

The plots of sensitivity and R$_{TF}$ for realistic detectors using different front shielding materials following carbon ion irradiation are presented in Fig. 10.

Thermal neutrons start to arrive at the detector in significant numbers after 10$^4$ ns. Due to their low kinetic energy, thermal neutrons do not directly contribute to false positives; however, they can cause the detector itself to become activated, contributing to an increase in background radiation, which, in turn, can result in the detection of false positives (depending on the wavelenths of emitted gamma radiation). As the detector ages, this problem will progressively increase; therefore, it is desirable to block thermal neutrons from the detector. Thermal neutrons can be almost entirely absorbed by a thin layer of certain materials with high thermal neutron cross sections, which will not significantly attenuate neutron capture gamma photon fluence—provided that the shielding material is distinct from the neutron capture isotope used during NCEPT treatment.

The addition of a thin layer of any of the evaluated shielding materials on the front face of the detector results in a very small decrease in $R_{TF}$ when using phantoms with a $^{10}$B neutron capture insert (Fig. 10a). This is a result of slightly increased attenuation and scatter of the neutron capture gamma photons prior to reaching the sensitive volume of the detector, while there is minimal impact on the background level of scattered photons, fast and intermediate neutrons. The same result is observed for both carbon and helium ion irradiation (see Supplementary Figure 4a).

In the case of the $^{157}$Gd neutron capture insert, the addition of boron or hafnium front shielding on the detector results in a substantial increase in $R_{TF}$ relative to the unshielded case (both for carbon and helium ion irradiation; see Supplementary Figure 4b), since the false positives are predominantly caused by neutron interactions within the detector (Fig. 10b). By contrast, the use of cadmium shielding with a $^{157}$Gd NCI in the target results in a very substantial decrease in $R_{TF}$, since the $^{113}$Cd neutron capture reaction results in the emission of high energy photons at 8.48 MeV and 9.04 MeV, which will cause single and double escape peaks to fall in the 7.94 MeV energy acceptance band^34^.

Since the addition of thermal neutron shielding almost entirely eliminates the problem of thermal neutron activation of the detector while having minimal negative impact on the sensitivity and specificity of the prompt gamma detector system (with the exception of cadmium shielding in the case of a $^{157}$Gd-bearing target), the use of thermal neutron shielding to prolong the life of the detector is justified; the best choice are cadmium for a $^{10}$B target and boron for the $^{157}$Gd target, both for carbon and helium ion beams.

## Conclusion

The feasibility of the proposed method for discriminating between $^{10}$B or $^{157}$Gd neutron capture events and other sources of prompt gamma radiation from the target volume during particle therapy, via energy windowing and temporal masking relative to the beam pulse arrival time, has been established. Overall, for targets containing a $^{10}$B NCI, the detector which obtained the highest $R_{TF}$ was the CdTe detector with a 60 ns timing mask and 1 µs irradiation duration. For the $^{157}$Gd NCI, the LSO detector provided the highest $R_{TF}$ with a 60 ns timing mask and 1 ms beam duration. The addition of a thin thermal-neutron shield to the front face of the detector results in a slight reduction in sensitivity and selectivity when using a boron NCI, however it allows almost all of the thermal neutrons to be absorbed before reaching the detector, avoiding the problem of neutron activation. In the case of the gadolinium NCI, the addition of front face shielding results in an increase in the $R_{TF}$ as the false positives are caused by neutron interactions within the detector.

## Supplementary Information

Supplementary Information.

## Data Availability

Full datasets generated and/or analysed during the current study are available from the corresponding author on reasonable request; sensitivity and selectivity results and additional timing and spectral plots are included as CSV files in the Supplementary Materials, with the CSV file format described in Section 4 of the Supplementary Materials PDF. Alternatively, all CSV files can be downloaded from the following repository: https://bitbucket.org/msafavi/promptgammadatabase.
