# Supplementary material for: Detection and discrimination of neutron capture events for NCEPT dose quantification
Source: Sci Rep. 2022 Apr 7;12:5863. doi: 10.1038/s41598-022-09676-x (PMC8990023; doi:10.1038/s41598-022-09676-x)
Supplement: Supplementary file 1 — Supplementary Information. [file 41598_2022_9676_MOESM1_ESM.pdf]

Prompt gamma based detection and discrimination of neutron capture events for NCEPT dose quantification - Supplementary Material

Andrew Chacon[1], Marissa Kielly[1,2], Harley Rutherford[1,2], Daniel R. Franklin[3], Anita Caracciolo[4,5], Luca Buonanno[4,5], Ilenia D'Adda[4,5], Anatoly Rosenfeld[2], Susanna Guatelli[2], Marco Carminati[4,5], Carlo Fiorini[4,5], and Mitra Safavi-Naeini[1,2,*]

[1]Australian Nuclear Science and Technology Organisation (ANSTO)
[2]Centre for Medical Radiation Physics, University of Wollongong, Australia
[3]Faculty of Engineering & IT, University of Technology Sydney, Australia
[4]Dipartimento di Elettronica, Informazione e Bioingegneria, Politecnico di Milano, Milano, Italy
[5]Istituto Nazionale di Fisica Nucleare (INFN), Sezione di Milano, Milano, Italy
[*]mitras@ansto.gov.au

1 Comparison of detector materials

Plots comparing the different detector material sensitivity and R_{TF} are presented in Supplementary Figure 1 for realistic detector models irradiated by a helium ion beam. The results for helium are very similar to those presented in the main paper for carbon; the best detector materials are CdTe for the ^{10}B NCI and LSO for ^{157}Gd.

2 Comparison of irradiation durations

The plots of sensitivity and R_{TF} for the two best detector materials for the ^{10}B and ^{157}Gd NCIs following irradiation by a helium ion beam as a function of temporal mask duration for a range of irradiation times are presented in Supplementary Figure 2 (for all detected events) and 3 (for photons only). For the ^{10}B NCI, the optimal irradiation duration was 1 μs, while for ^{157}Gd, the situation is more complex. In this case, R_{TF} is maximised for irradiation duration of 10 μs, but this is at the cost of poor sensitivity. However, it is noted that for an irradiation duration of 1 ms (as for carbon ions), R_{TF} is still good (around 5) while the sensitivity remains high relative to other irradiation durations. Therefore, the 1 ms duration may be considered an optimal compromise for helium irradiation of a ^{157}Gd target.

3 Shielding materials

The plots of sensitivity and R_{TF} for realistic detectors using different front shielding materials following helium ion irradiation are presented in Supplementary Figure 4. The best-performing shielding material for ^{10}B is cadmium, while for ^{157}Gd the best-performing material is boron.

4 Full Dataset

The full dataset is available for download. The filenames use the following structure:

beam_detectorMaterial_shielding_pmmaSOBP-300x300x300-**NCI_fraction|sensitivity_ideal|realistic**.csv

where

- **beam** - is the beam type:
  - 2-4 (helium); or
  - 6-12 (carbon)
- **detectorMaterial** - is the material of the detector - either:
  - CdTe (^{10}B NCI only);

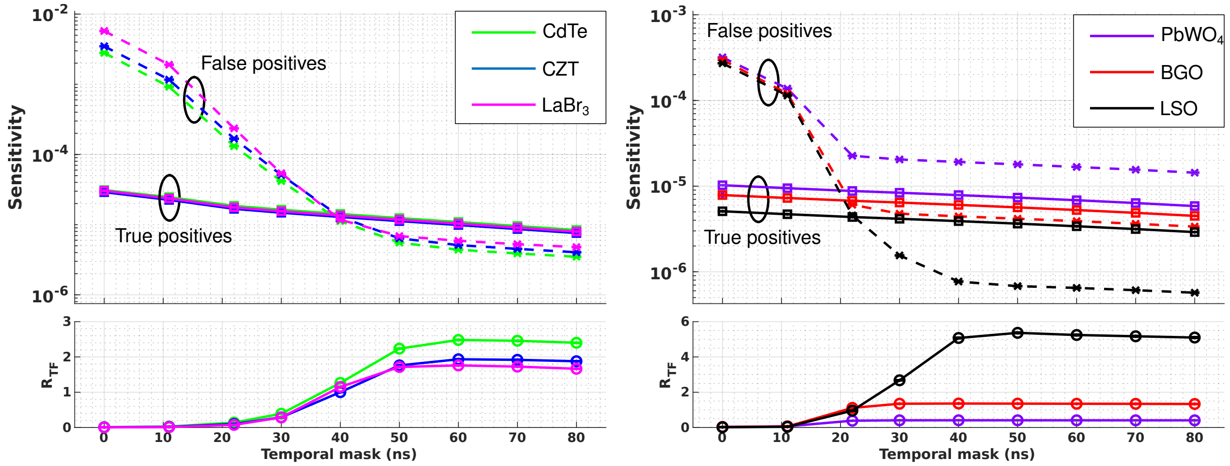

(a) 1000 ns irradiation; ^{10}B NCI

(b) 1 ms irradiation; ^{157}Gd NCI

Supplementary Figure 1. Sensitivity (upper plot) and R_{TF} (lower plot) of events detected using different detector materials following irradiation by a helium ion beam. For the ^{10}B NCI, magenta denotes the $LaBr_3$ detector, green the CdTe detector and blue the CZT detector. For ^{157}Gd NCI, red denotes the BGO detector, black the LSO detector and purple the $PbWO_4$ detector. In the upper plots in each subfigure, square markers (□) joined by unbroken lines denote true positives, while cross markers ($\times$) joined by dashed lines denote false positives.

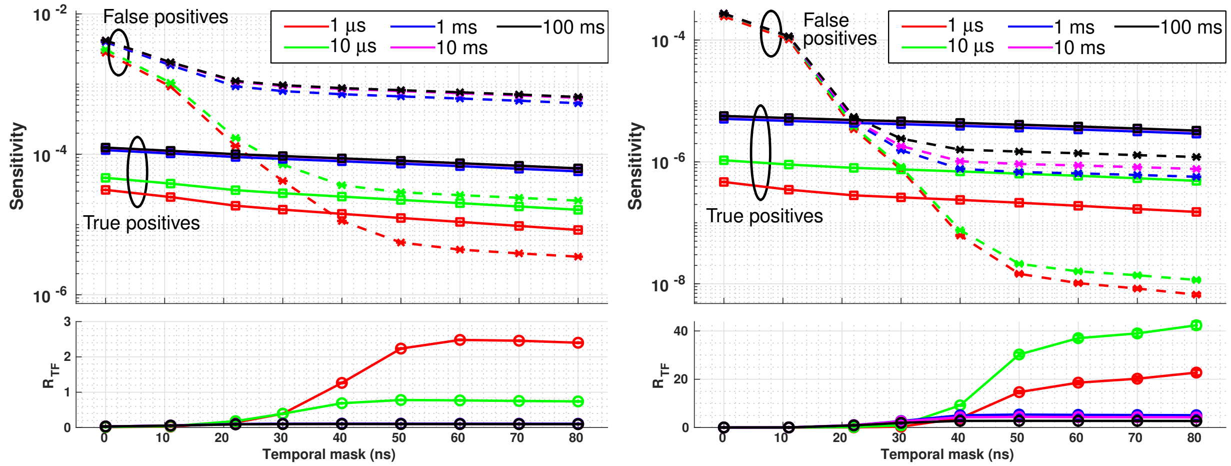

(a) ^{10}B NCI; CdTe detector

(b) ^{157}Gd NCI; LSO detector

Supplementary Figure 2. Sensitivity (upper plot) and R_{TF} (lower plot) for all detected events following target irradiation by a helium ion beam as a function of temporal mask duration for a range of different total irradiation periods. In all plots, the red markers denote a total irradiation time of 1 μs, green 10 μs, blue 1 ms, magenta 10 ms and black 100 ms. In the upper plots in each subfigure, square markers (□) joined by unbroken lines denote true positives, while cross markers ($\times$) joined by dashed lines denote false positives.

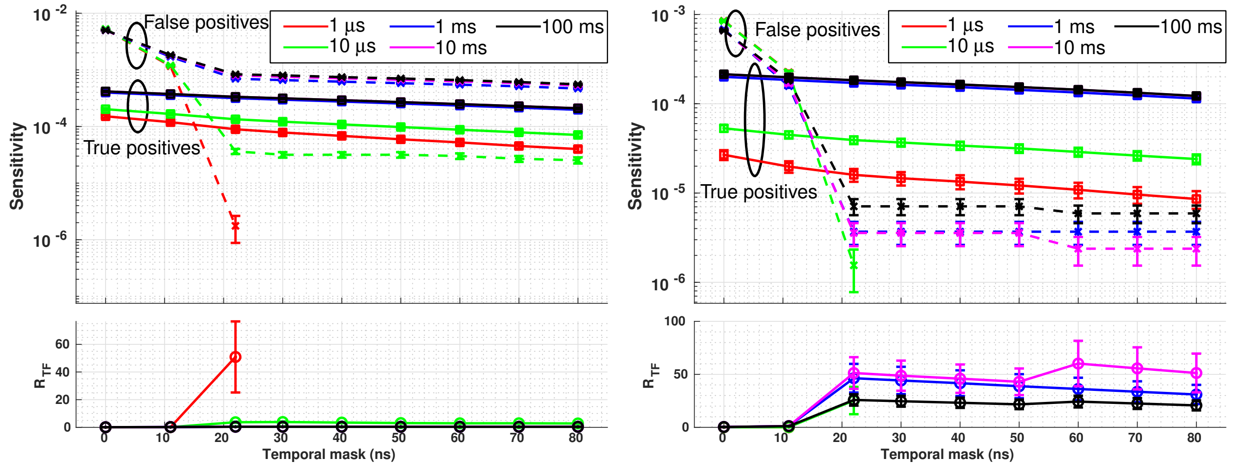

(a) ^{10}B NCI; CdTe detector

(b) ^{157}Gd NCI; LSO detector

Supplementary Figure 3. Sensitivity (upper plot) and R_{TF} (lower plot) for photon detections only following target irradiation by a helium ion beam as a function of temporal mask duration for a range of different total irradiation times. In all plots, the red markers denote an irradiation time of 1 μs, green 10 μs, blue 1 ms, magenta 10 ms and black 100 ms. In the upper plots in each subfigure, square markers (☐) joined by unbroken lines denote true positives, while cross markers ($\times$) joined by dashed lines denote false positives.

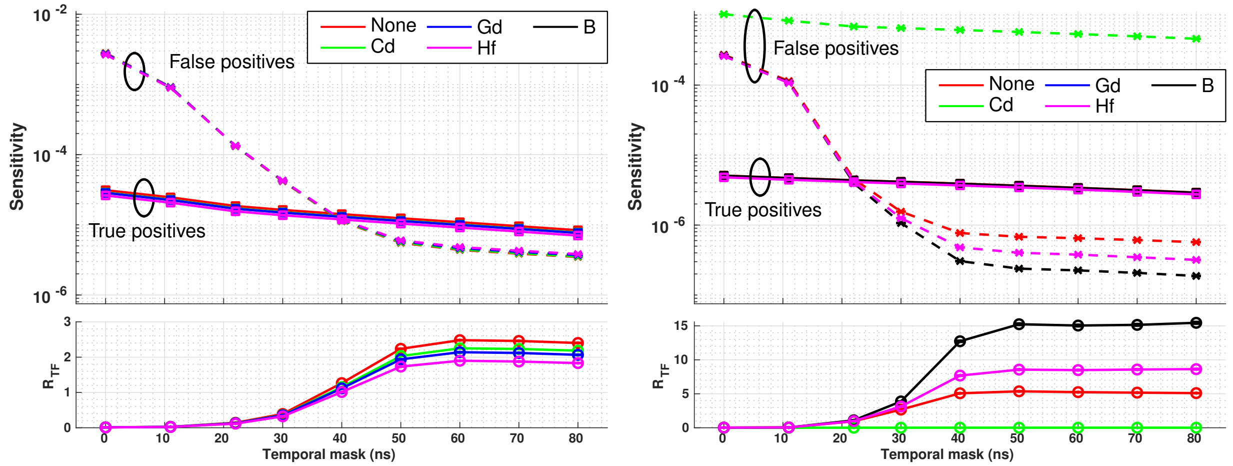

(a) 1000 ns helium ion beam; ^{10}B NCI

(b) 1 ms helium ion beam; ^{157}Gd NCI

Supplementary Figure 4. Sensitivity (upper plot) and R_{TF} (lower plot) for events recorded in a detector shielded by different neutron-absorbing materials, following target irradiation by a helium ion beam. In all plots, the red markers denote an unshielded detector, green cadmium shielding, blue gadolinium shielding (^{10}B NCI only), black boron shielding (^{157}Gd NCI only), and magenta hafnium shielding. In the upper plots in each subfigure, square markers (☐) joined by unbroken lines denote true positives, while cross markers ($\times$) joined by dashed lines denote false positives.

- CZT (^{10}B NCI only);
- LaBr3 (^{10}B NCI only);
- PbWO4 (^{157}Gd NCI only);
- BGO (^{157}Gd NCI only); or
- LSO (^{157}Gd NCI only).

- **shielding** - is the front shielding material:
  - unshielded;
  - Cd_1_mm;
  - Hf_1_mm;
  - Gd_1_mm (^{10}B NCI only); or
  - B_1_mm (^{157}Gd NCI ony).
- **NCI** - is the neutron capture insert used: either
  - B10; or
  - Gd157
- **fraction|sensitivity** - specifies whether the data is:
  - sensitivity - normalised to number of photons incident on the detector; or
  - fraction - normalised so that the sum of true/false positive/negatives equals 1.
- **ideal|realistic** - specifies whether the data is:
  - ideal - from the ideal detector; or
  - realistic - the data is from the realistic detector.

For example, the file name for the fraction of true/false positive/negatives using an ideal detector and helium beam with a ^{157}Gd NCI, with a BGO detector, using 1 mm of boron shielding and a ^{157}Gd NCI is:

`2-4_BGO_B_1mm_pmmaSOBP-300x300x300-Gd157_fraction_ideal.csv`

The data in each CSV file is organised into blocks according the the combination of temporal mask duration and irradiation interval, with either overall sensitivity to different event classes (true and false positives and negatives) or the fraction of overall detected events of each type listed. Temporal masks are 0 ns, 11 ns, 22 ns, 30 ns, 40 ns, 50 ns, 60 ns, 70 ns and 80 ns, and irradiation intervals are either 1 μs, 10 μs, 1 ms, 10 ms, or 100 ms, for a total of 45 combinations of mask and irradiation interval for each set of simulation conditions.
